# Testing a compact mobile air purification unit, does it do the job

**DOI:** 10.1186/2047-2994-4-S1-P52

**Published:** 2015-06-16

**Authors:** P De Waegemaeker, J Boelens, I Leroux-Roels

**Affiliations:** 1 Hospital Infection Control, Ghent University Hospital, Ghent, Belgium; 2 Medical Microbiology, Ghent University Hospital, Ghent, Belgium

## Introduction

The use of mobile air purification systems in hospitals is widely accepted and even promoted by the CDC, but turns out to be quite expensive. In this study, the Hospital Infection Control Team (HICT) of the Ghent University Hospital evaluated a more cost-effective mobile HEPA filter unit.

## Objectives

To determine whether a compact, more cost effective, mobile HEPA filter unit was able to purify the air significantly from airborne particles and fungal spores.

## Methods

A mobile HEPA filter unit ‘FilterQueen Defender’ (FQD, HMI industries) was installed in the centre of a small room (36 m³). A particle counter (HHPC-6 airborne particle counter, Art instruments) and a microbiological air sampler (MAS100, Merck) were set up in the corners of that room.

Measurements were conducted during several hours every 15 to 30 minutes, before and after activation of the FQD. After deactivation, post-measurements were performed as part of a validity check. The 3 different settings of the FQD, corresponding to an increasing ventilator speed, were evaluated during the test.

## Results

In this test, the number of airborne particles measured were clearly and positively influenced by the FQD in ventilator speed settings 1 and 2. Both smaller particles (≥ 0.5µ) as well as bigger particles (≥ 5.0µ) were reduced to less than 10% (and in some cases down to 1%) of their initial load. There was however no measurable difference between settings 2 and 3. Before activation of the FQD, microbiological air sampling showed presence of fungal spores in the air (6 to 17 cfu/m³, in 8 of 8 measurements). After activation of the FQD, the number of fungal spores was reduced to zero within 1 hour.

## Conclusion

Despite its compactness and significantly lower price (80% less as compared to standard mobile HEPA filters), the FQD has shown to be highly effective in purifying air, as expressed by the lower particle count and the absence of fungal spores within 1 hour.

Based upon this experience we decided to purchase multiple FQD devices that are now routinely used throughout the hospital in high risk settings, such as during construction works or to prevent airborne infection in isolation rooms.

## Disclosure of interest

None declared.

